# Indole derivatives ameliorated the methamphetamine-induced depression and anxiety via aryl hydrocarbon receptor along “microbiota-brain” axis

**DOI:** 10.1080/19490976.2025.2470386

**Published:** 2025-02-25

**Authors:** Xi Wang, Miaoyang Hu, Weilan Wu, Xinyu Lou, Rong Gao, Tengfei Ma, S Thameem Dheen, Jie Cheng, Jianping Xiong, Xufeng Chen, Jun Wang

**Affiliations:** a Center for Global Health, The Key Laboratory of Modern Toxicology, Ministry of Education, Department of Toxicology, School of Public Health, Nanjing Medical University, Nanjing, China; b Department of Anatomy, Yong Loo Lin School of Medicine, National University of Singapore, Singapore, Singapore; c Department of Hygienic Analysis and Detection, The Key Laboratory of Modern Toxicology, Ministry of Education, School of Public Health, Nanjing Medical University, Nanjing, China; d Stem Cell and Neural Regeneration and Key Laboratory of Cardiovascular & Cerebrovascular Medicine, School of Pharmacy, Nanjing Medical University, Nanjing, China; e Department of Emergency Medicine, The First Affiliated Hospital of Nanjing Medical University, Nanjing, Jiangsu, China

**Keywords:** Depression and anxiety, methamphetamine, indole derivatives, aryl hydrocarbon receptor

## Abstract

In addition to the high neurotoxicity, depression, and anxiety are the most prominent characteristics of methamphetamine (Meth) withdrawal. Studies to date on the issue of Meth-associated depression and anxiety are focused on the brain, however, whether peripheral homeostasis, especially the “microbiota-gut” axis participates in these adverse outcomes, remains poorly understood. In the current study, with the fecal microbiota transplantation (FMT) assay, the mice received microbiota from Meth withdrawal mice displayed marked depression and anxiety behaviors. The 16S rRNA sequencing results showed that Meth withdrawal contributed to a striking reduction of *Akkermansia, Bacteroides, Faecalibaculum, Desulfovibrio, and Anaerostipes*, which are known to be associated with tryptophan (TRP) metabolism. Noteworthily, the substantial decreases of the indole derivatives from the TRP metabolic pathway, including IAA, IPA, ILA, IET, IArA, IAld, and TRM were observed in the serum of both Meth abusing humans and mice during Meth withdrawal with the UHPLC-MS/MS analysis. Combining the high and low TRP diet mouse model, the mice with high TRP diet obviously impeded Meth-associated depression and anxiety behaviors, and these results were further strengthened by the evidence that administration of IPA, IAA, and indole dramatically ameliorated the Meth induced aberrant behaviors. Importantly, these protective effects were remarkably counteracted in aryl hydrocarbon receptor knockout (AhR KO) mice, underlining the key roles of microbiota-indoles-AhR signaling in Meth-associated depression and anxiety. Collectively, the important contribution of the present work is that we provide the first evidence that peripheral gut homeostasis disturbance but not limited to the brain, plays a key role in driving the Meth-induced depression and anxiety in the periods of withdrawal, especially the microbiota and the indole metabolic disturbance. Therefore, targeting AhR may provide novel insight into the therapeutic strategies for Meth-associated psychological disorders.

## 1. Introduction

According to the 2023 World Drug Report, the number of individuals who used amphetamine-type stimulants reached as high as 36 million,^1^ and the number of Meth abusers is still increasing. As a typical representative of amphetamine-type drugs, Meth (commonly known as “ice”) is highly addictive and neurotoxic, with an average elimination half-life approximately to 10 hours in individuals. Compared to the traditional drugs, Meth abuse is more prone to causing severe psychological disorders, particularly depression and anxiety, and the depressive symptoms often emerge during Meth withdrawal period.^2^ A cross-sectional study reported a depression prevalence rate of 39.5% among 1,277 participants,^3^ while another cohort study indicated that 157 out of 243 participants (64.6%) exhibited depressive symptoms during Meth withdrawal, with 74 (30.5%) experiencing moderate and 83 (34.1%) experiencing severe depression.^4^ Meth abuse inflicts severe physical and psychological harm, such as self-harm and suicidal behaviors, posing significant challenges to public health. Therefore, alleviating Meth withdrawal-related depressive symptoms and deciphering the underlying mechanisms is imperative.

For Meth-induced depression and anxiety, more attention is paid to the central nervous system (CNS), while few studies have tried to unravel the cellular basis of Meth-induced psychological disorders from peripheral systems. As a “second brain,” the gut and its interactions with microbiota are implicated in CNS activities, including mood, stress response, and cognitive function through neural, endocrine, immune, and metabolic pathways,^5–7^ therefore, targeting gut microbiota for the treatment of psychological disorders might be a promising therapeutic approach. The gut-brain axis is a complex bidirectional communication network that links the gut microbiota to the CNS. Tryptophan (TRP), one of the nine essential amino acids, plays a crucial role in coordinating gastrointestinal function and CNS activities. TRP is primarily metabolized through three pathways: the kynurenine pathway, the serotonin pathway, and the microbial metabolic pathway (Indole metabolic pathway).^8^ For the microbial metabolic pathway, the gut microbiota metabolizes TRP into several indole derivatives, such as indole-3-aldehyde (IAld), indole-3-acetic acid (IAA), indole-3-propionic acid (IPA), indole-3-pyruvate (IPYA), etc.^9^ In recent years, accumulating reports pointed to the influence of the TRP metabolic pathway far beyond the traditional focus on the study of the serotonin and the kynurenine pathways, the TRP microbial metabolic pathway has gained more and more attention since the metabolites indole and its derivatives are recognized as the essential signaling molecules within the microbiota-gut-brain axis, capable of influencing brain function and behaviors.^10^ In depression patients, the levels of indole and its derivatives have been shown to correlate closely with the severity of depressive and anxiety symptoms.^11^ For instance, *Bifidobacterium breve CCFM1025* effectively alleviates depression and related gastrointestinal disorders in patients, concomitant with the increased levels of IPA,^12^ which has been demonstrated to inhibit CNS inflammation.^13^ However, whether the microbiota and (or) the corresponding metabolites participated in driving the Meth-induced depression and anxiety, to date, remain largely unknown.

Aryl hydrocarbon receptor (AhR), a transcriptional factor expressed in immune cells, microglia, intestinal epithelial cells, etc., can be activated by specific small molecules from diet, microbial metabolites, and environmental pollutants.^14,15^ Upon binding with its ligands, AhR translocates to the nucleus and forms a heterodimer with ARNT to regulate CYP1A1, CYP1A2, and CYP1B1 expression,^16^ a negative feedback loop that is crucial for indole and its derivatives degradation. A study reported that by colonizing *Escherichia coli* modified for indole metabolism and TRP metabolism-deficient *Escherichia coli*, indole, a TRP metabolite of intestinal flora, and its derivatives can activate neurogenesis through the AhR pathway.^17^ Additionally, intermittent fasting may promote axonal regeneration after sciatic nerve crush injury in mice by enhancing IPA production by gut Gram-positive bacteria.^18^ Indole has been found to harbor the neuroprotective properties both in vitro and in vivo,^19^ since supplementation with indole and its metabolites such as IS, IPA and IAld significantly reduces CNS inflammation in germ-free mice.^13^ Nonetheless, whether the indole metabolic pathway is involved in Meth-induced depression and anxiety remains ambiguous. Thus, deciphering the roles of indole derivatives and AhR signaling in Meth-induced neurobehavioral changes is of significant importance.

Therefore, in the present study, the Meth withdrawal mouse model was established to evaluate the effects of Meth on gut microbiota composition, TRP metabolic pathways, and the interaction with AhR. Additionally, a Meth mouse model supplemented with indole derivatives and AhR agonist, in combination with AhR KO mice were used, aiming to elucidate the critical roles of the AhR, which may serve as a bridge between the gut microbiota and the CNS, particularly in the regulation of microglial morphology and neurogenesis. Moreover, the levels of the indole metabolites in the serum of the Meth withdrawal individuals were also evaluated, which may provide solid support for the disturbance of the indole metabolic pathway in Meth-induced depression and anxiety.

## 2. Materials and methods

### 2.1. Reagents and antibodies

Methamphetamine (purity over 99%) was provided by the National Institutes for Food and Drug Control (Beijing, China). Standard substances IAA (CAS: 87-51-4), IPA (CAS: 830-96-6), IArA (CAS: 29953-71-7), IAM (CAS: 879-37-8), IPYA (CAS: 392-12-1), IAld (CAS: 487-89-9), TRM (CAS: 61-54-1), IET (CAS: 526-55-6), and IND (CAS: 120-72-9) were purchased from Sigma-Aldrich (Darmstadt, Germany). ILA (CAS: 1821-52-9) was purchased from Macklin (Shanghai, China). Internal standards Trp-D5 and IAA-D5 were obtained from Toronto Research Chemicals (Toronto, Canada). HPLC-grade methanol, acetonitrile, formic acid, and 6-Formylindolo[3,2-b] carbazole (Ficz) were purchased from Sigma-Aldrich (Darmstadt, Germany). Anti-DCX (4604S) was purchased from Cell Signaling Technology (MA, USA). Anti-IBA1 (019–19741) was purchased from WAKO (Tokyo, Japan). Anti-IBA1 (ab5076) was obtained from Abcam (Cambridge, UK). Alexa Fluor™ 488 goat anti-rabbit IgG (A-11008) was purchased from Thermo Fisher Scientific (Waltham, MA, USA). The HRP-conjugated secondary antibodies were from Biosharp (Nanjing, China).

### 2.2. Animals and treatment

Male wild-type (WT) C57BL/6 mice (6 weeks old, 15–20 g) were purchased from the Experimental Animal Center of Nanjing Medical University and maintained in an SPF environment with a controlled room temperature (RT, 21–23°C), humidity (50–60%) and 12 h/12 h light-dark cycle. AhR^−/−^ C57BL/6 mice were obtained from GemPharmatech Co., Ltd (Nanjing, China). All animal experiments in this study were approved by the Experimental Animal Welfare Ethics Review Committee of Nanjing Medical University (approval No. IACUC-2302024).

#### 2.2.1. Meth administration

After 1 week of acclimatization, the mice were randomly divided into control and Meth withdrawal groups (5 mg/kg, i.p., once daily for 10 consecutive days, followed by a 14-day withdrawal, after that named “WD 14d”). The control group received saline injections.

#### 2.2.2. Fecal microbiota transplantation (FMT) treatment

A gut microbiota depletion mouse model was constructed with a one-week antibiotic cocktail (ABX) treatment, consisting of vancomycin (50 mg/kg/day), neomycin (100 mg/kg/day), metronidazole (100 mg/kg/day), ampicillin (100 mg/kg/day), and amphotericin B (1 mg/kg/day). All antibiotics were purchased from Solarbio Science & Technology Co., Ltd. (Beijing, China). The depletion of endogenous gut microbiota was assessed by aerobic/anaerobic culturing on TSA plates. Fresh fecal samples from Meth-withdrawal donor mice were collected, weighed, and suspended in sterile PBS at a 1:10 ratio (w/v, feces/PBS). The suspension was vortexed, then centrifuged at 1000 rpm for 5 minutes at 4°C. The supernatant was collected and centrifuged at 8000 rpm for 5 minutes at 4°C to remove bacterial pellets. The final mixture was resuspended in sterile PBS for transplantation. Recipient mice (including FMT-Control, Saline, and FMT-WD 14d groups) were administered 200 μL fecal suspension via oral gavage every two days for 14 consecutive days.

#### 2.2.3. Dietary TRP supplementation

The TRP feed was purchased from TROPHIC Animal Feed High-Tech Co., Ltd (Nantong, China) and the mice were randomly divided into four groups: Control (Saline), Low TRP (0.09% TRP), Meth withdrawal (WD 14d), and Meth withdrawal with high TRP diet (WD 14d+High TRP, 1.19% TRP). Mice in the control and Meth withdrawal groups were fed a control diet (AIN93M standard diet for rodents).

#### 2.2.4. Indoles supplementation and Ficz treatment

The indoles solution (a mixture of IAA, IPA, and indole) was prepared with a final concentration of 300 μM before being dispensed into drinking bottles. The AhR agonist, Ficz (Sigma-Aldrich; USA) was dissolved by DMSO (Sigma-Aldrich, USA) and subsequently diluted with olive oil (Sigma-Aldrich). The solution was administered via intraperitoneal injection (i.p.) at a dosage of 0.04 mg/kg/day, thrice weekly.

### 2.3. Population samples

This study employed a rigorous cross-sectional design, recruiting a total of 78 Meth abusers and 79 healthy controls. The sample size was determined using G Power^20^ based on an a priori power analysis, which calculated the required sample size to achieve a statistical power of 0.8 with a medium effect size (Cohen’s d = 0.5) and a significance level of 0.05 (Figure S5). All participants provided informed consent for sample collection and participation in the study. The study protocol was approved by the Medical Ethics Committee of Changshu First People’s Hospital, affiliated with Suzhou University (Ethical Application and Approval 2016–01). Serum samples from individuals with a history of Meth abuse within 1 month and the serum of Meth abusers and healthy controls were collected for analysis.

### 2.4. Assessment of depression- and anxiety-like behaviors

Mouse depression-like behaviors were assessed by the tail suspension test (TST) and forced swim test (FST), while anxiety-like behaviors were evaluated by the open field test (OFT) and elevated plus maze (EPM) test. For a detailed protocol, please refer to the Supplementary Materials.

### 2.5. 16S rRNA sequencing and analysis

Fecal samples were collected from mice at 8:00–10:00 AM on WD 0d and WD 14d. The samples were placed into sterile tubes, and immediately frozen in liquid nitrogen. Total microbial DNA was extracted using the QIAamp DNA Stool Mini Kit (Qiagen, Hilden, Germany) according to the manufacturer’s instructions. For bacterial 16S rRNA gene sequencing, the V3-V4 hypervariable regions of the 16S rRNA gene were amplified using Premix Ex Taq™ Hot Start Version (Takara, Dalian, China) with the following universal primers: 319F (5′-ACTCCTACGGGAGGCAGCAG-3′) and 806 R (5′-GGACTACHVGGGTWTCTAAT-3′). Amplicons were purified using the AxyPrep DNA Gel Extraction Kit (Axygen Biosciences, Union City, CA, USA) and quantified with QuantiFluor-ST (Promega, USA). The purified amplicons were then pooled in equimolar concentrations and subjected to library preparation. The quality and quantity of the amplicon library were assessed before sequencing. The prepared libraries were sequenced on an Illumina MiSeq platform (Illumina Inc., San Diego, CA, USA) to generate paired-end reads. Sequencing data were processed and analyzed using BMKCloud (www.biocloud.net), where bioinformatics pipelines including quality filtering, operational taxonomic unit (OTU) clustering, and taxonomic classification were applied to assess microbial diversity and composition across different experimental groups.

### 2.6. UHPLC-MS/MS analysis of indole derivatives

The sample preparation of serum and fecal samples was optimized based on previous studies.^21^ In brief, after preparing the serum and fecal samples, the samples were analyzed using an ExionLC™ AC HPLC system coupled to a SCIEX Triple Quad 5500 Plus mass spectrometer (AB SCIEX, USA). The mass spectrometer was operated in positive electrospray ionization mode and multiple reaction monitoring (MRM) mode. Data were acquired by Analyst® software (AB SCIEX, USA) and analyzed by MultiQuant™ software (AB SCIEX, USA).

### 2.7. Immunofluorescence and microglial morphometric analysis

Mice were perfused with 4% paraformaldehyde, and the brain tissue was collected and fixed in 4% paraformaldehyde. After dehydration, the tissue was embedded in an optimal cutting temperature (OCT) compound and cryosectioned in liquid nitrogen after freezing. Coronal brain sections of the hippocampus were obtained using a cryostat maintained at −15°C to −20°C, with a section thickness of 25–30 μm. The sections were blocked with a blocking solution containing 5% goat serum, then permeabilized with 0.3% Triton X-100. Afterward, the primary antibodies were incubated overnight, followed by incubation with secondary antibodies. After washing, the sections were mounted with DAPI (ab104139, Abcam). Photomicrographs were acquired using a Zeiss LSM900 scanning laser confocal microscope (Zeiss, Oberkochen, Germany). The 3D reconstruction of microglia was imaged on the confocal laser scanning microscope with Z-stacks completed in the z-axis direction with a step size of 1.0 μm. Microglial morphology was recorded and analyzed using IMARIS software (version 9.0.1, Bitplane, Switzerland). 5–6 cells were reconstructed for each mouse, with 3–4 mice per group.

### 2.8. Quantitative RT-PCR

Total RNA was extracted from brain tissue lysates using the TRIzol reagent (343903, Life Technologies, USA). The concentration and purity of the extracted RNA were assessed using a NanoDrop 2000 spectrophotometer (Thermo, USA). SYBR Green Supermix (11201ES08, Yeasen Biotech Co., Ltd., China) was used to measure relative mRNA levels. Fluorescence changes were tracked using a LightCycler 480 system (Roche, Switzerland), with GAPDH serving as the reference gene. The primer sequences of RT-PCR are detailed in Supplementary Materials Table S1.

### 2.9. Histological analysis

Colon tissues were fixed in formalin, embedded in paraffin, and sectioned into 4 μm-thick slices for histopathological analysis. These sections were stained with hematoxylin and eosin (H&E), then scanned using a Pannoramic scanner (3DHISTECH, Germany), and histological scores were determined according to previous methods.^22^

### 2.10. Microscale thermophoresis (MST)

The MST assay was performed based on the previous study.^23^ IPA, IAA and indole were diluted to eight different concentrations and mixed in equal amounts, and the concentration of GFP-AhR was determined using a GFP quantification kit (abcam, ab235672, USA). The mixed samples were loaded into capillaries (Monolith NT.115 Capillary, NanoTemper, Germany) and the thermophoresis signals were measured using a Monolith NT.115 instrument (NanoTemper, Germany) following the manufacturer’s standard protocol. Fluorescence thermophoresis and changes in kd values were analyzed using Nano Temper Analysis software (Germany).

### 2.11. Flow cytometry

Mice were anesthetized and perfused with ice-cold PBS to remove circulating leukocytes. After perfusion, the hippocampus was gently dissociated and placed in an RPMI digestion solution containing 1 mg/mL type II collagenase (Sigma, USA) and 1% DNAse (Sigma, USA), followed by incubation at 37°C for 45 minutes. After digestion, myelin was removed using a 30% Percoll solution in RPMI. The isolated cells were washed, incubated with an Fc receptor-blocking solution for 5 minutes, and then incubated with antibodies. Microglia were identified as CD45^mid^CD11b^+^ cells. All antibodies were purchased from eBioscience (Thermo Fisher Scientific, USA).

### 2.12. Statistical analysis

Statistical analyses were performed using GraphPad Prism 8.0 (San Diego, CA, USA). Most data are presented as mean ± SEM, except for box plots, which display maximum and minimum values. Statistical significance was defined as *p* < 0.05. The Shapiro-Wilk test and Levene’s test were used to assess normality and homogeneity of variance, respectively. For comparisons between two independent groups that met these assumptions, an unpaired Student’s t-test was employed, while the Mann-Whitney U test was used for non-normally distributed data. For multiple group comparisons, one-way or two-way analysis of variance (ANOVA) was applied as appropriate, followed by Tukey’s post hoc test for pairwise comparisons due to its high efficiency in analyzing datasets with equal or similar sample sizes. For 16S rRNA sequencing data, principal coordinate analysis (PCoA) was used to evaluate the main compositional features of the samples. Differential microbial taxa between the two groups were identified using the Wilcoxon rank-sum test due to the non-normal distribution of the data. For multiple-group comparisons, the Kruskal-Wallis test followed by the Benjamini-Hochberg procedure for false discovery rate correction was employed. Receiver operating characteristic (ROC) curve analysis was performed using SPSS 22.0 (Chicago, IL, USA) to differentiate Meth abusers from healthy controls based on indole derivative levels.

## 3. Results

### 3.1. Meth-induced depression and anxiety behaviors and microbiota disturbance in mice

**Figure 1.**
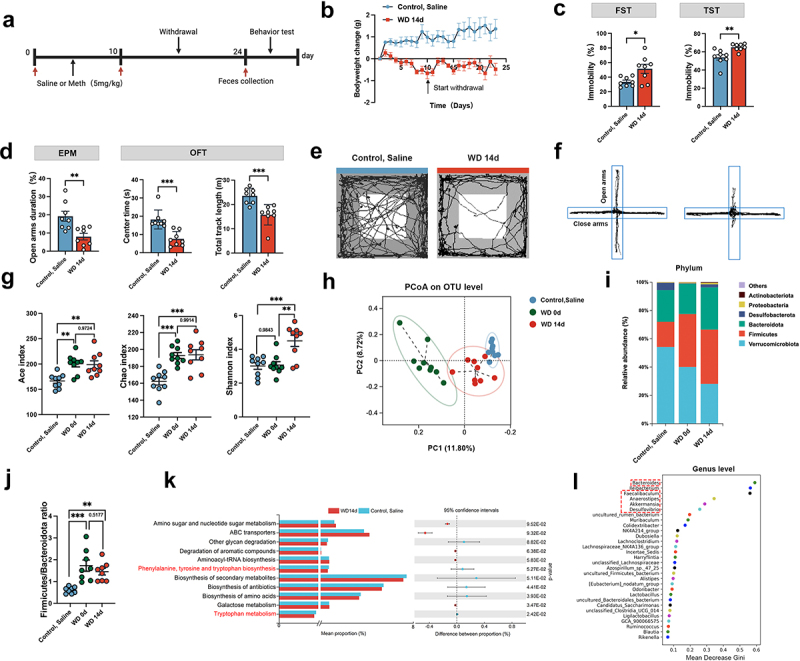
Effects of Meth on mouse behaviors and gut microbiota. (a) Experimental design and timeline. (b) Body weight change (*n*=8–10 per group). (c,d) Immobility time (%) in the FST and TST; time spent in the open arms (%) in the EPM test; time spent in the center (s) and total track length (m) in the OFT (*n*=8 per group). (e,f) The representative trajectory diagrams in the OFT and EPM. (g)Ace, Chao, and Shannon index of α diversity. (h) Principal coordinate analysis of β diversity based on Bray-Curtis distances among the control, saline, WD 0d, and WD 14d groups. (i) Stacked chart of gut microbiota composition at the phylum level. (j) The ratio of phylum *Firmicutes*/*Bacteroidetes*. (k) Genomic functional prediction of gut microbiota community by Tax4Fun. (l) Random forest analysis of gut microbiota importance at the genus level on WD 14d and control, saline groups. Each dot indicates an individual mouse. Data are expressed as mean ± SEM, *p < 0.05, **p < 0.01, ***p < 0.001.

Since microbiota dysbiosis influences depression and anxiety behaviors,^24^ it is logical to explore whether Meth exposure impacts microbiota and the behaviors. To this end, the microbiota changes and the psychological behaviors induced by Meth were examined. With the daily intraperitoneal injection of Meth (5 mg/kg) for 10 days and the following 14-day withdrawal, the mouse fecal samples were collected on day 10 and day 24, then by behavioral testing (Figure 1(a)). During the Meth exposure and withdrawal periods, mice exhibited a significant decrease in body weight compared to the control group (Figure 1(b)). In parallel, the withdrawal mice displayed significant depression behaviors, with increased immobility states in both of the TST and FST. Additionally, the mice spent less time in the center arena in OFT and open arms in the EPM test, characterized as anxiety behaviors (Figure 1(c–f)). To gain a more comprehensive knowledge of the behavioral changes induced by Meth, the composition of the gut microbiota was analyzed. The alpha diversity was assessed by calculating the Ace, Chao, and Shannon indices at the OTU level. It showed that the Ace and Chao indices of the WD 0d and WD 14d groups were significantly higher than those of the control group, and the Shannon index of the WD 14d group was higher than that of the control groups albeit the WD 0d group exhibited no obvious changes (Figure 1(g)). According to principal coordinate analysis (PCoA), the beta diversity of the gut microbiota differed among the control, WD 0d, and WD 14d groups (Figure 1(h)). For microbiota abundance, the relative abundance of *Firmicutes* significantly increased whereas the abundance of *Verrucomicrobia* dramatically decreased in the WD 0d and WD 14d groups (Figure 1(i)). Additionally, the ratio of *Firmicutes* to *Bacteroidetes* (Figure 1(j)) obviously increased in WD 0d group, even lasting 14 days post Meth treatment, reflecting a stable state of dysbiosis. Furthermore, the Random Forest analysis ranked the importance of the microbiota on WD 14d and the control group at the genus level. Tax4Fun was employed to evaluate the functional changes in the gut microbiota. The analysis revealed that the phenylalanine, tyrosine, and tryptophan biosynthesis, as well as the tryptophan metabolism (highlighted in red), exhibited significant differences between the WD14d and control groups, with a notable reduction in activity observed in the WD14d group. Additionally, pathways such as ABC transporters, other glycan degradation, and degradation of aromatic compounds also demonstrated significant variations between the two groups (Figure 1(k)). Based on the mean decrease in the Gini coefficient, the top six genera contributing to group differences were *Bacteroides*, *Ileibacterium*, *Faecalibaculum*, *Anaerostipes*, *Akkermansia*, and *Desulfovibrio* (Figure 1(l)).

### 3.2. Meth withdrawal dramatically decreased indole derivatives metabolized by the gut microbiota

**Figure 2.**
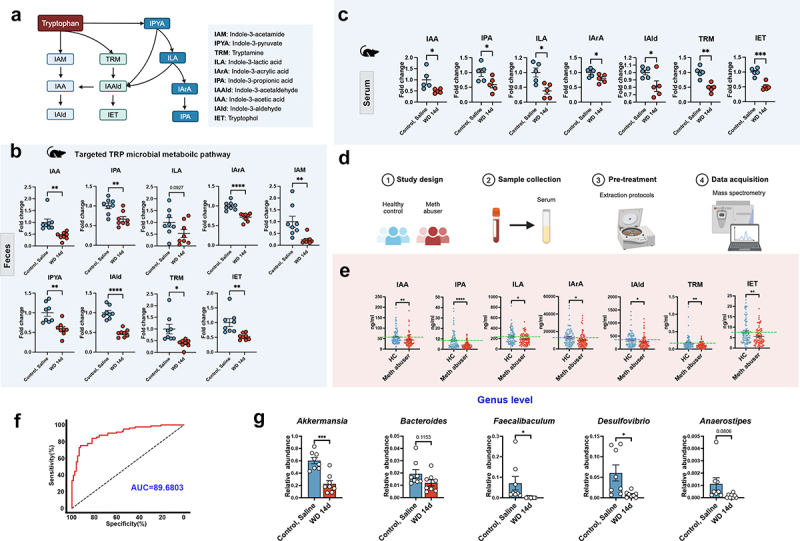
Indole metabolism in Meth withdrawal mice and humans. (a) Schematic representation of the TRP microbial metabolic pathway, including IAA, IPA, ILA, IArA, IAM, IPYA, IAld, TRM and IET. (b) Analysis of indole derivatives in feces of WD 14d mice compared to control mice (*n* = 8 per group). (c) Analysis of indole derivatives in serum samples in mice. (d) Schematic of sample collection, pre-treatment, and mass spectrometry detection and analysis in healthy controls and Meth abusers. (e) Comparison of indole derivatives in the serum of Meth abusers versus healthy controls (HC) (*n* = 78–80 per group). (f) ROC curve for distinguishing Meth abusers from healthy controls based on indole derivative levels. (g) Relative abundance of gut microbiota genera in WD 14d and control mice. Each dot indicates an individual mouse. Data are expressed as mean ± SEM, *p < 0.05, **p < 0.01, ***p < 0.001, ****p < 0.0001.

To further tie together the microbiota dysbiosis and the depression and anxiety behaviors induced by Meth, the indole metabolic pathway (Figure 2(a)) was detected in mouse feces and serum. Of note, the levels of most indole metabolites, including IAA, IPA, ILA, IArA, IAM, IPYA, IAld, TRM and IET were significantly reduced in the WD 14d group compared with the control group, implying a blockage in indole metabolism (Figure 2(b,c)). To verify whether the aberrant indole metabolism was also displayed in humans, the levels of indole metabolites in the serum of human healthy controls (HC) and Meth abusers were examined (Figure 2(d)). Consistently, the levels of indole derivatives, including IAA, IPA, ILA, IArA, IAld, TRM and IET were strikingly decreased in Meth abusers (Figure 2(e)). Noteworthily, the receiver operating characteristic (ROC) curve displayed a high capacity in distinguishing healthy controls from Meth abusers, with an AUC value of 89.6803 (Figure 2(f)). Interestingly, with the 16S RNA sequence assay, the microbiota known to metabolize TRP into indole derivatives, including *Akkermansia*, *Bacteroides*, *Faecalibaculum*, *Desulfovibrio*, and *Anaerostipes* were markedly reduced in the WD 14d group (Figure 2(g)). Among these taxa, *Akkermansia* was significantly and consistently reduced following Meth exposure, prompting further investigation into its functional role in TRP metabolism. To validate the contribution of *A. muciniphila* to indole metabolism, we assessed its ability to metabolize TRP into indole derivatives. Using the *A. muciniphila* MucT (ATCC BAA-835) strain, we demonstrated that *A. muciniphila* could metabolize TRP into multiple indole derivatives, including IPA, IAld, IArA, and TRM, in a concentration-dependent manner (Figure S1). These findings provide direct evidence linking the reduction of *A. muciniphila* to the decrease in indole derivatives observed during Meth withdrawal. Collectively, these results suggest that a pronounced blockage of indole metabolism via the reduced indole-producing microbiota by Meth.

### 3.3. Transplantation of feces from Meth-withdrawal mice resulted in depression- and anxiety-like behaviors in recipient mice

**Figure 3.**
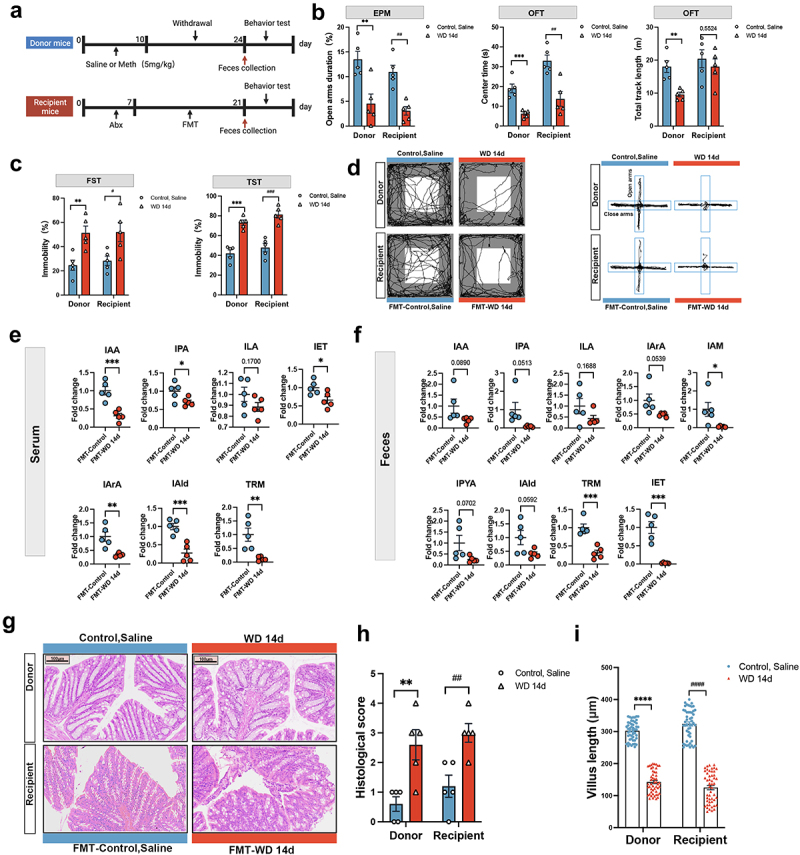
Roles of the microbiota in mediating Meth-induced anxiety and depression-like behavior in mice. (a) Experimental design and timeline. (b,c) Time spent in the open arms (%) in the EPM test; and time spent in the center (s) and total track length (m) in the OFT; immobility time (%) in the FST and TST. (d)The representative trajectory diagrams in the OFT and EPM. (e,f) Levels of indole derivatives in the serum and feces of FMT recipient mice. (g-h) H&E staining of the colons (scale bar 100 μm) and the histological score analysis. (i) Villus length analysis. Data are expressed as mean ± SEM, *p < 0.05, **p < 0.01, ***p < 0.001,****p < 0.0001; ^#^*p* < 0.05, ^##^*p* < 0.01, ^###^*p* < 0.001, ^####^*p* < 0.0001.

Having determined the Meth induced microbiota dysbiosis, we hypothesized whether microbiota dysbiosis can induce anxiety- and depression-like changes. Herein, the fecal microbiota transplantation (FMT) experiment was performed. Figure 3(a) presents the experimental timeline, dividing the mice into two groups: Donor and Recipient mice. The donor mice were treated with either Meth or saline, followed by behavioral testing and fecal sample collection on the last day of withdrawal (WD14d). For recipient mice, the gut microbiota was depleted with an antibiotic cocktail (ABX), then received FMT from the donor mice. To address the potential concern regarding residual Meth or its metabolites in donor fecal material, we performed UHPLC-MS/MS analysis to detect Meth levels in fecal samples at different time points post-injection. Meth was detectable in the feces 2 hours after injection. However, no residual Meth was detected in feces collected at 1 day, 3 days, or 14 days post-injection. These results confirm that the effects observed in FMT-WD 14d mice were not attributable to residual Meth in donor fecal material but rather to changes in the gut microbiota composition (Figure S3). To further explore the mechanisms underlying Meth-induced gut dysbiosis, we conducted supplementary experiments focusing on intestinal barrier integrity and mucus production. Our results revealed that Meth withdrawal (WD 14d) significantly increased serum LPS levels, indicating compromised gut barrier function (Figure S4(a)). Tight junction-related genes (*ZO1*, *Occludin*, *Claudin5*) were significantly downregulated in WD 14d mice, consistent with barrier dysfunction (Figure S4(b)). Additionally, goblet cell numbers were markedly reduced (Figure S4(c)), accompanied by a significant decrease in MUC2 expression and mucus layer thickness, as demonstrated by immunofluorescence and Alcian blue staining (Figures S4(d) and S4(e)). These disruptions in the mucus layer are particularly relevant to the observed reduction in *Akkermansia* abundance, as this bacterium depends on mucin as its primary nutrient source. The reduction in mucus production and goblet cell numbers likely deprives *Akkermansia* of its ecological niche, leading to its decline during Meth withdrawal. This loss of *Akkermansia*, in turn, may exacerbate gut barrier dysfunction and further contribute to gut dysbiosis. Behavioral assessments indicated that the WD 14d donor mice and the recipient mice (FMT-WD 14d) exhibited significant anxiety- and depression-like behaviors, characterized by reduced time spent in the open arms of the EPM and the center of the OFT, decreased total distance traveled in the OFT, and increased immobility in the FST and TST (Figure 3(b–d)). Furthermore, the indole derivatives in the serum and feces of recipient mice significantly reduced (Figure 3(e,f)), indicating that Meth-induced behavioral changes might be partially ascribed to the microbiota dysbiosis. Since the impairments of the intestinal integrity were linked to the psychiatric disorders, the histological analysis of the intestinal structure in both donor and recipient mice was conducted. It showed that the pathological scores were significantly increased and the villus length was notably reduced in the WD 14d and FMT-WD 14d mice (Figure 3(g–i)), further supporting the critical role of gut microbiota and the corresponding metabolites in Meth-induced anxiety and depression-like behaviors.

### 3.4. Dietary TRP improves the aberrant behaviors and metabolism in Meth withdrawal mice

**Figure 4.**
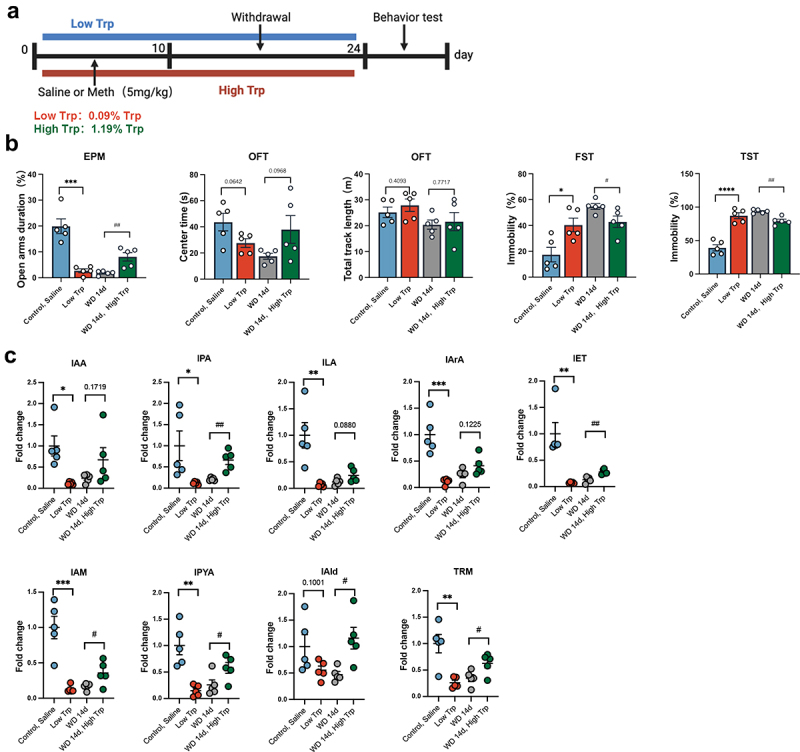
Effects of dietary TRP supplementation on Meth-induced behavioral changes and levels of indole derivatives in mice. (a) Experimental design and timeline. (b) Time spent in the open arms (%) in the EPM test; time spent in the center (s) and total track length (m) in the OFT; immobility time (%) in the FST and TST. (c) Levels of indole derivatives in fecal samples from different groups. Data are expressed as mean ± SEM, *p < 0.05, **p < 0.01, ***p < 0.001, ****p < 0.0001; ^#^*p* < 0.05, ^##^*p* < 0.01.

Having determined the reduction of indole metabolites and the corresponding producing microbiota, then we explored whether dietary TRP supplementation could reverse the Meth-induced aberrant behaviors and indole metabolic changes. Figure 4(a) shows the timeline of the experimental design. The high TRP diet (1.9% TRP) was pretreated and maintained until Meth withdrawal for 14 days, and the low TRP diet (0.09% TRP) was applied here for positive control aiming to decipher the roles of the reduced indole metabolites in affecting behaviors. It showed that mice in the low TRP diet group and the WD 14d group exhibited significant anxiety-like changes, manifesting as the reduction of open-arm residence time in the EPM, and a decreased residence time in the center area accompanied by a decreased total trajectory length in the OFT test. Meanwhile, the low TRP diet stimulated pronounced depression like changes due to the increased immobility time in the FST and TST. Agree with our assumption, the high TRP diet substantially ameliorated the Meth induced anxiety and depression-like behaviors (Figure 4(b)), in parallel, it retarded the Meth-induced decreased levels of IAA, IPA, ILA, IArA, IET, IAM, IPYA, IAld and TRM. Particularly the levels of IPA, IAM, IPYA, and TRM (Figure 4(c)), suggesting a salutary role of TRP metabolites in alleviating Meth-induced anxiety and depression-like behaviors. To further substantiate the causal link between the reduction in indole derivatives and AhR pathway activity, we performed qPCR analysis of AhR-related genes. Our results revealed that both the low TRP diet group and the WD 14d group exhibited significant downregulation of *Ahr*, *Cyp1a1*, and *Cyp1b1*, indicating suppression of the AhR pathway. Importantly, supplementation with the high TRP diet reversed this suppression, restoring gene expression levels to those comparable to the control group (Figure S2). These findings confirm that the reduction in indole derivatives observed in vivo contributes to AhR pathway dysregulation and suggest that dietary TRP supplementation mitigates these effects by restoring indole derivative levels and AhR signaling activity.

### 3.5. Meth withdrawal causes microglial dystrophy and reduces neurogenesis in the hippocampus

**Figure 5.**
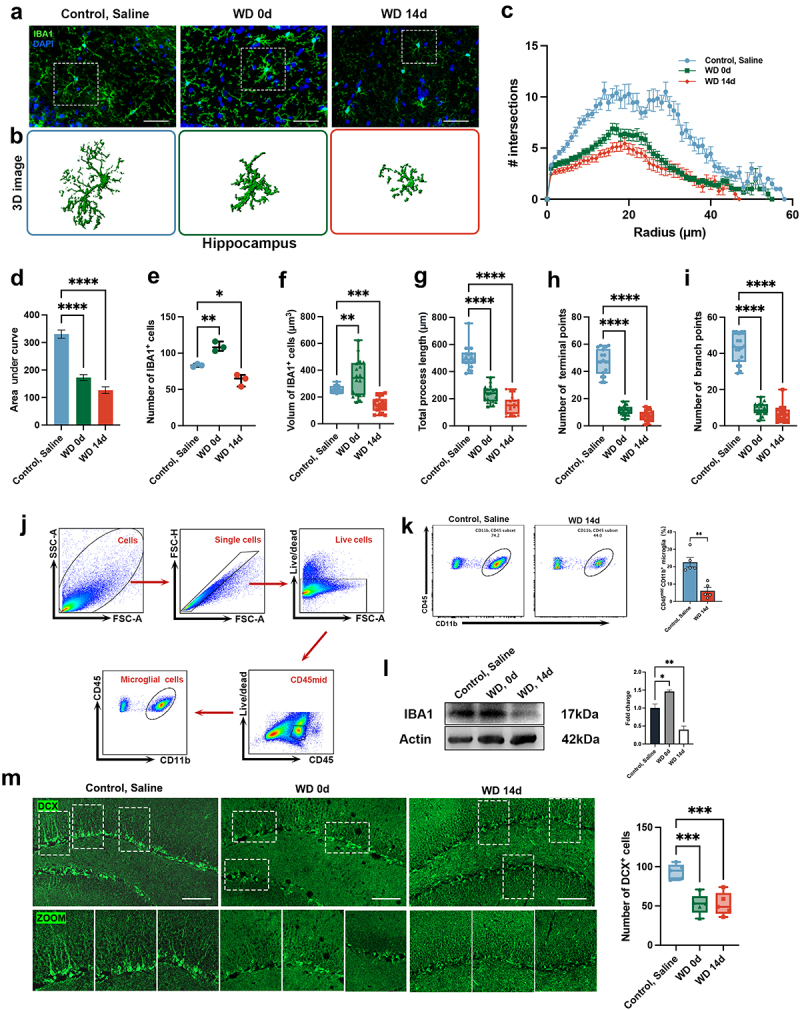
Effects of Meth on microglial morphology and neurogenesis in the hippocampus. (a) Representative immunofluorescence images of IBA1^+^ microglia (green) in the hippocampus of control, saline, WD 0d, and WD 14d groups. DAPI (blue) was used for nuclei staining (scale bar 50 μm). (b) 3D reconstructions of microglial cells from the hippocampal regions of each group. (c) Sholl analysis quantifies the number of microglial intersections as a function of the distance from the cell soma. (d-i) Quantitative analyses of microglial morphology: (d) area under the curve (AUC) from Sholl analysis, (e) number of IBA1^+^ cells, (f) volume of IBA1^+^ cells, (g) total process length, (h) number of terminal points, and (i) number of branch points. (j) Flow cytometry gating strategy for identifying microglial cells (CD11b^+^ CD45^mid^) in the hippocampus. (k) Flow cytometry results quantifying the percentage of CD11b^+^ CD45^mid^ microglia in the control, saline, and WD 14d groups. (l) Western blot analysis of IBA1 protein expression in hippocampal lysates. (m) Immunofluorescence staining for doublecortin (DCX, green) in the hippocampus (scale bar 100 μm), with zoomed-in images highlighting neurogenesis. Data are expressed as mean ± SEM, *p < 0.05, **p < 0.01, ***p < 0.001, ****p < 0.0001.

To further decipher the adverse impact of Meth on mouse behaviors, the microglial morphology and neurogenesis in the hippocampus were analyzed. As depicted in Figure 5(a), the microglia undergo dramatical morphological changes after the Meth challenge, the WD 0d microglia exhibited an increased cell volume, shorter and thicker processes, and reduced branching. These features indicated the activated microglia in the early stage of Meth exposure. The presentation of the 3D reconstructions of these microglia (Figure 5(b)), visually illustrated these morphological changes. However, by WD 14d, microglial processes were significantly reduced, characterized by the dystrophic cells, including the retracted and fragmented branches. Sholl analysis quantitatively validated these changes, which showed a gradual decrease in microglial branches from WD 0d to WD 14d (Figure 5(c–d)). Furthermore, the morphological parameters of microglia were quantified, including process length, branch, and terminal points. It showed a pronounced reduction in microglial branches after Meth exposure, and these changes did not store even after 14 days of withdrawal (Figure 5(g–i)). The number of IBA1^+^ cells increased in the WD 0d group, along with enlarged cell volume, but significantly decreased in number and volume by WD 14d (Figure 5(e–f)). The flow cytometry and Western blot analyses further confirmed the reduction in microglial numbers in the WD 14d group (Figure 5(j–l)). Additionally, DCX, a marker of the immature neuron and neuronal development, was used for assessing neurogenesis. As depicted in Figure 5(m), a significantly decreased number of DCX^+^ cells in the DG region of both WD 0d and WD 14d mice was observed, indicating neurogenesis defects. These results suggested that Meth exposure dynamically modulated microglial outcome over time and disturbed neurogenesis in the hippocampus, which might be involved in Meth-induced anxiety and depression-like behaviors.

### 3.6. Indole derivatives drive AhR activation and alleviate Meth-induced anxiety and depression-like behaviors

**Figure 6.**
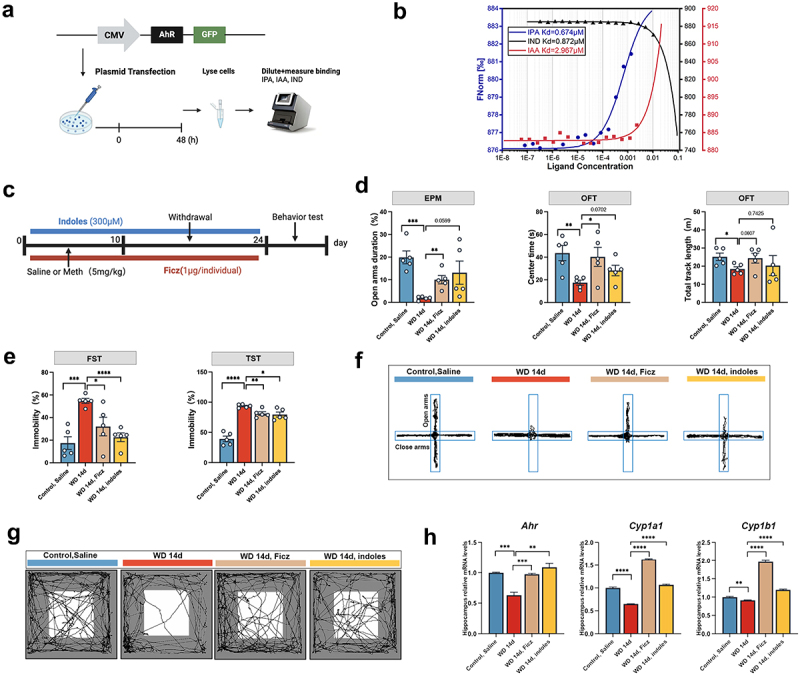
Indole derivatives and AhR agonist Ficz alleviated Meth-induced anxiety and depression-like behaviors. (a) Schematic diagram of the MST experimental design to measure the binding of IPA, IAA, and IND to AhR. (b) The binding affinity of IPA, IND, and IAA to AhR. (c) Experimental design and timeline. (d,e) Time spent in the open arms (%) in the EPM test; time spent in the center (s) and total track length (m) in the OFT; immobility time (%) in the FST and TST (*n*=5 per group). (f,g) the representative trajectory diagrams in the OFT and EPM. (h) mRNA expression of AhR pathway-related genes (*Ahr*, *Cyp1a1*, and *Cyp1b1*) in the hippocampus. Data are expressed as mean ± SEM, *p < 0.05, **p < 0.01, ***p < 0.001, ****p < 0.0001.

To ascertain whether the salutary effects of TRP were ascribed to its metabolites, the interaction between indole derivatives and AhR was examined. Since some indole derivatives are the natural ligands for AhR, the binding affinity of IPA, IAA, and IND to AhR was determined by the MST assay, which was based on the dramatical reduction of these indole derivatives in serum and feces in Meth treated mice. As shown in Figure 6(a), IPA, IAA, and IND exhibited different binding affinities to AhR, with Kd values of 0.674 µM, 2.967 µM, and 0.872 µM, respectively, indicating that these indole derivatives can effectively bind to AhR (Figure 6(b)). Then we moved to address whether the salutary effects of TRP were ascribed to indole derivatives and AhR interaction. Figure 6(c) presents the experimental timeline, which included four groups: Control, WD 14d, WD 14d + Ficz, and WD 14d + indoles. Consistent with our assumption, exogenous administration of indole derivatives (IPA, IAA, and IND) markedly alleviated the Meth induced depression- and anxiety-like behaviors, evidenced by reduction of the immobility time in FST and TST, paralleled with increased time spent in the open arms of the EPM and in the center area of the OFT (Figure 6(d–j)). For addressing the roles of AhR in Meth induced depression- and anxiety-like changes, Ficz, one potent agonist of AhR, was administrated. Consistent with the results presented in indole derivatives administration, Ficz treatment obviously mitigated the Meth induced depression- and anxiety-like changes, unraveling the critical roles of AhR in this process.

Additionally, the downstream signaling of the AhR was examined as well. Compared with the control group, the mRNA levels of the *Ahr*, *Cyp1a1*, and *Cyp1b1* were significantly reduced in the WD 14d group, an effect that was substantially rescued by both Ficz and indole derivatives supplementation, implying the pivotal roles of the AhR pathway in alleviating Meth-induced behavioral abnormalities (Figure 6(h)).

### 3.7. The protective effects of indole derivatives and AhR against Meth-induced microglial morphological dystrophy and neuronal damage

**Figure 7.**
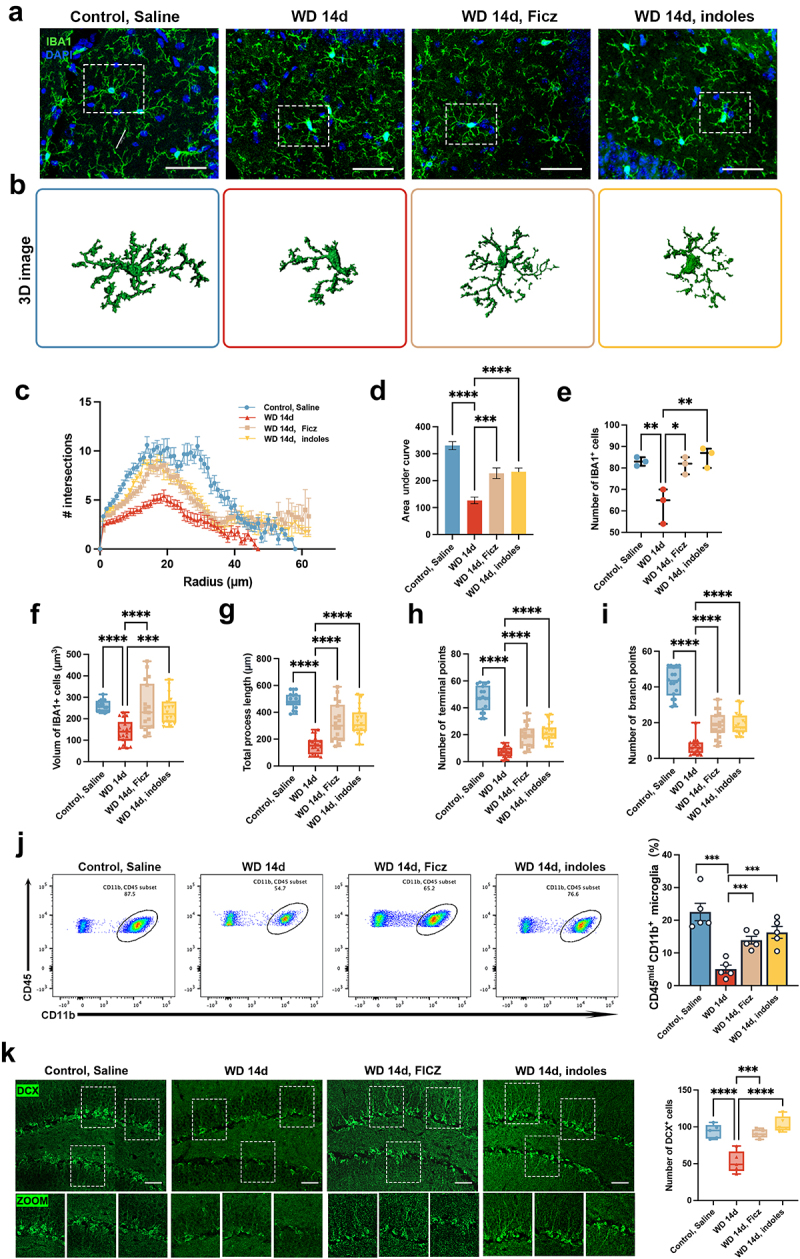
Protective effects of indole derivatives and Ficz on Meth-induced microglial morphological changes and neurogenesis defects in the hippocampus. (a) Representative immunofluorescence images of IBA1^+^ microglia (green) in the hippocampus of control, saline, WD 14d, WD 14d+Ficz, and WD 14d+indoles groups. DAPI (blue) was used for nuclei staining (scale bar 50 μm). (b) 3D reconstructions of microglial cells in the hippocampus. (c) Sholl analysis quantifies the number of microglial intersections as a function of the distance from the cell soma. (d-i) quantitative analyses of microglial morphology: (d) AUC from Sholl analysis, (E) number of IBA1^+^ cells, (f) volume of IBA1^+^ cells, (g) total process length, (h) number of terminal points, and (i) number of branch points. (j) Flow cytometry quantifies the percentage of CD11b^+^ CD45^mid^ microglia in each group. (k) Immunofluorescence staining for DCX (green) in the hippocampus of each group, with zoomed-in images highlighting neurogenesis (scale bar 100 μm). Data are expressed as mean ± SEM, *p < 0.05, **p < 0.01, ***p < 0.001, ****p < 0.0001.

Having confirmed the effects of indole derivatives and the AhR signaling pathway on Meth-induced behavioral abnormalities, then we sought to investigate the impact of these molecules on microglial morphology and neurogenesis, which are closely associated with depression and anxiety. As shown in Figure 7(a), the microglial morphology underwent dramatical changes after Meth withdrawal, including decreased process length and branch numbers, and these phenomena were obviously restored after administration of Ficz and indole derivatives. The 3D reconstructed images in Figure 7(b) visually depicted these morphological changes, emphasizing the remodeling effects of Ficz and indole derivatives on microglia. Meanwhile, the Sholl analysis indicated a significant reduction in microglial branching complexity in the WD 14d group, while supplementation with Ficz or indole derivatives obviously restored this complexity (Figure 7(c)). Further quantification of microglial morphological parameters showed that in the WD 14d group, the area under the curve (AUC, Figure 7(d)), IBA1^+^ cell number (Figure 7(e)), cell volume (Figure 7(f)), process length (Figure 7(g)), terminal points (Figure 7(h)), and branch points (Figure 7(i)) were all significantly reduced, indicating the marked damages in microglial morphology and function. Of note, Ficz and indole derivatives markedly reversed the aforementioned damages. Flow cytometry analysis indicated that Ficz and indole derivatives significantly increased the proportion of CD11b^+^CD45^mid^ microglia in the WD 14d group, supporting the restorative effects of Ficz and indole derivatives on microglia (Figure 7(j)). Similarly, Ficz and indole derivatives notably ameliorated the Meth induced impaired neurogenesis, evidenced by the increased DCX^+^ cells (Figure 7(k)).

### 3.8. AhR KO impedes the salutary effects of indole derivatives against the Meth induced depression and anxiety-like behaviors

**Figure 8.**
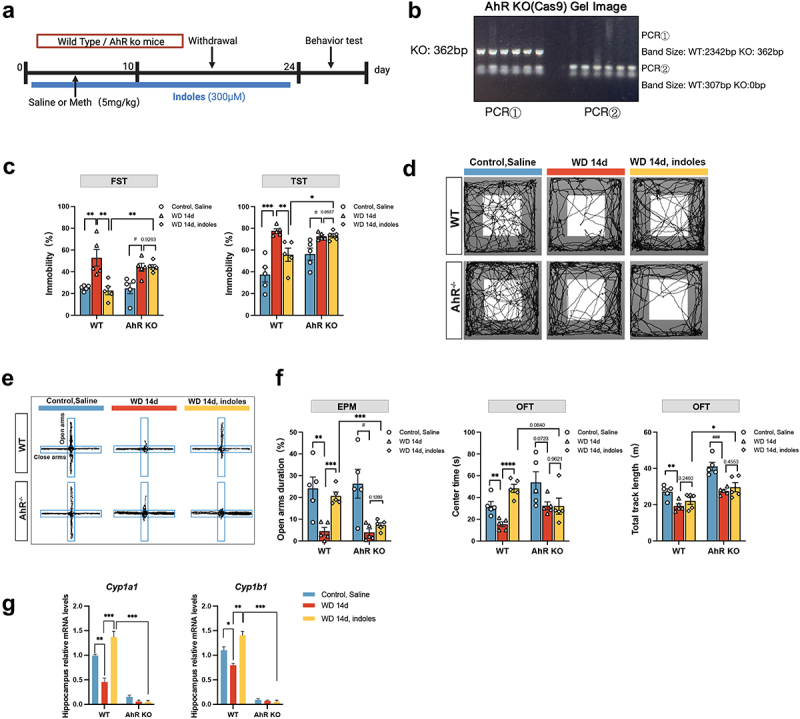
Roles of AhR KO in Meth-induced depressive and anxiety-like behaviors. (a) Experimental design and timeline. (b) Gel image showing PCR results confirming the AhR KO in mice. (c) Immobility time (%) in the FST and TST. (d,e) The representative trajectory diagrams in the OFT and EPM. (f) Time spent in the open arms (%) in the EPM test; time spent in the center (s) and total track length (m) in the OFT. (g) mRNA expression of AhR-related gene (*Ahr*, *Cyp1a1*, and *Cyp1b1*) in the hippocampus. Data are expressed as mean ± SEM, *p < 0.05, **p < 0.01, ***p < 0.001, ****p < 0.0001; ^#^*p* < 0.05, ^###^*p* < 0.001.

To confirm the underlying roles of the AhR in the anti-depression and anti-anxiety of indole derivatives in the Meth withdrawal mouse, the AhR knockout (AhR KO) mouse was applied. Figure 8(a) presents the experimental timeline of Meth exposure and indole supplementation in both wild-type (WT) and AhR KO mice and the behavioral tests were conducted after the withdrawal period. The confirmation of AhR KO mice was confirmed by PCR, which showed the absence of the AhR gene (Figure 8(a)). The behavioral analysis revealed that in the WD 14d group, WT mice exhibited significant depression-like behaviors, as indicated by increased immobility time in the FST and TST, and these effects were obviously alleviated by indole derivatives, noteworthily, the protective effects of indole derivatives were abolished in AhR KO mice (Figure 8(c)). Similarly, the beneficial effects of indole derivatives on anxiety were notably diminished in AhR KO mice as well (Figure 8(d–f)). mRNA analysis further supported the activation of AhR signaling by indole derivatives, since indole derivatives significantly restored the decreased levels of *Cyp1a1*, and *Cyp1b1* genes induced by Meth, and these effects were ablated in AhR KO mice. These results, taken together, underscore the critical roles of AhR in indole derivative-mediated behaviors and molecular signalings (Figure 8(g)).

### 3.9. AhR KO hampers the protective effects of indole derivatives against Meth induced microglial dystrophy and neurogenesis defects

**Figure 9.**
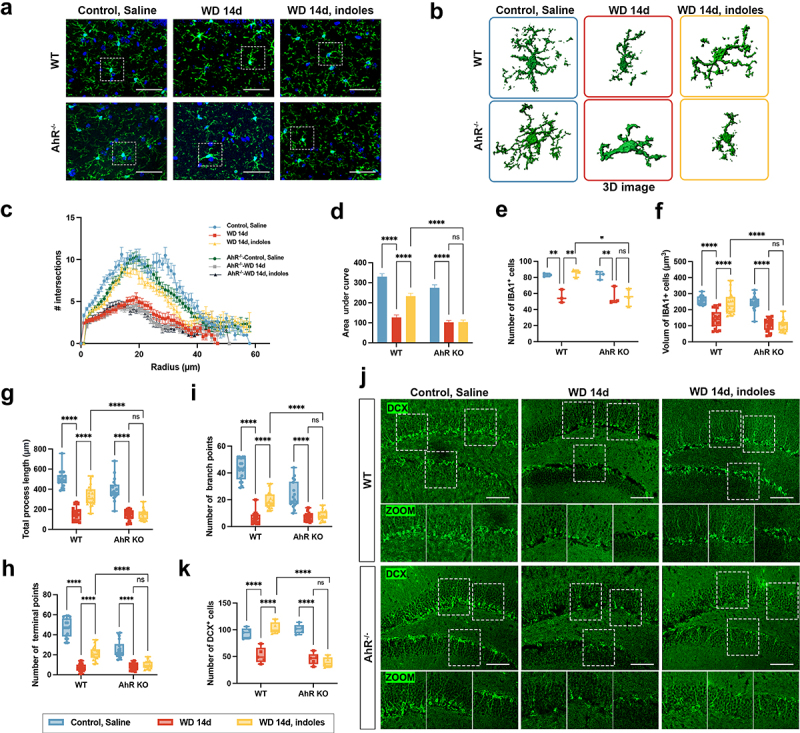
AhR KO alleviated the protective effects of indole derivatives against Meth-induced microglial dystrophy and neurogenesis defects. (a) Representative immunofluorescence images of IBA1^+^ microglia (green) in the hippocampus. DAPI (blue) was used for nuclei staining (scale bar 50 μm). (b) 3D reconstructions of microglial cells from the hippocampal regions of each group. (c) Sholl analysis quantifies the number of microglial intersections as a function of the distance from the cell soma. (d–i) Quantitative analyses of microglial morphology: (d) area under the curve (AUC) from Sholl analysis, (e) number of IBA1^+^ cells, (f) volume of IBA1^+^ cells, (g) total process length, (h) number of terminal points, and (i) number of branch points. (j) Immunofluorescence staining for DCX (green) in the hippocampus, with zoomed-in images highlighting neurogenesis (scale bar 100 μm). Data are expressed as mean ± SEM, *p < 0.05, **p < 0.01, ***p < 0.001, ****p < 0.0001.

Since the critical roles of the indole derivative in the modulation of the microglial morphology and neurogenesis aforementioned, then we investigated whether the effects were dependent on the AhR. As shown in Figure 9, the morphology of microglia (Figure 9(a)) and 3D reconstructed images (Figure 9(b)) in the hippocampal region were significantly changed in WT and AhR KO mice. A significant reduction in branching complexity was observed in the WD 14d group, which was obviously restored following indole derivative supplementation. These protective effects, however, were markedly attenuated in AhR KO mice (Figure 9(c)), indicating the crucial role of AhR signaling in microglial morphological remodeling. Further quantification of morphological parameters revealed pronounced decreases of AUC, IBA1^+^ cell number, cell volume, process length, terminal branch number, and branch points in the WD 14d group, whereas indole derivative treatment significantly restored these deteriorated impacts. Specifically, the beneficial effects were abolished in AhR KO mice (Figure 9(d–i)), supporting the importance of AhR signaling in the regulation of microglial morphology. Similarly, the protective effects of the indole derivatives against the Meth induced neurogenesis defects were restrained in AhR KO mice, since the DCX^+^ cells in the Meth+indole derivative were markedly decreased in AhR KO mice (Figure 9(k)). These findings underscore the pivotal roles of AhR in the remodeling of microglial morphology and neurogenesis in Meth-treated mice, unraveling the neuroprotective effects of indole derivatives via AhR.

## 4. Discussion

Epidemic studies have found that Meth withdrawal can trigger depression,^25–27^ and similar results were validated in Meth-withdrawal mice.^28–30^ The distribution in distinct tissues of Meth post-exposure in mice was examined and its levels in the blood and brain approached zero at 4 and 8 hours, respectively.^2^ Thus, the inflammatory effects and depressive behaviors induced by Meth withdrawal may represent a continuation of the adverse effects on distinct tissues and organs after initial exposure.

The toxicity of Meth to date has predominantly concentrated on the central nervous, circulatory, and respiratory systems, while less attention has been paid to the digestive system. As the “second brain” and the most predominant immunol system, the intestinal system may be highly susceptible to Meth attack. Therefore, the psychiatric disorders induced by Meth may not only limit to its direct impact on the brain, and the microbiota-gut axis may presumably be the major target. Studies have shown that Meth exposure alters the α and β diversity of gut microbiota in mice, increasing the relative abundance of pathogenic microbiota and reciprocally decreasing the beneficial ones.^31^ Additionally, microbiota abundance changes in Meth-exposed mice were significantly correlated with depression-like behaviors and neurotoxicity.^32^ In recent years, the roles of gut microbiota and the corresponding metabolites in regulating host behaviors and brain function have garnered considerable attention, particularly the TRP microbial metabolites, which are considered crucial to the regulation of the gut-brain axis. Thus, the current study aims to address the alterations of gut microbiota and the corresponding metabolites, especially the potential roles of indole derivatives in Meth-induced anxiety and depression-like behaviors in mice. In line with our assumption, Meth withdrawal significantly altered the microbiota composition in mice, and transplantation of the gut microbiota from Meth-exposed mice to recipient mice induced anxiety- and depression-like behaviors, providing evidence for the involvement of microbiota in the pathology. Intriguingly, Meth withdrawal resulted in a marked reduction in the levels of the indole metabolite-producing microbiota, thereby inhibiting the TRP microbial metabolic pathway and decreasing the indole derivative levels in serum and feces. Reciprocally, a high-TRP diet, which facilitates the restoration of indole derivative levels, significantly ameliorated the anxiety- and depression-like behaviors in Meth withdrawal mice. Importantly, we corroborated these findings in a population of Meth abusers, whose levels of indole derivatives were obviously reduced, suggesting the involvement of the gut microbiota and its indole metabolites in the Meth withdrawal period in humans.

TRP, an essential amino acid and the only amino acid containing the indole structure, is primarily obtained from diet. Numerous in vivo and in vitro studies have demonstrated that *Akkermansia* influences gut health and modulates immune responses by metabolizing TRP into indole derivatives, including indole, IAA, IArA, and ILA.^33–35^ Similarly, *Bacteroides* species are particularly active in the distal colon, where they ferment TRP to produce indole derivatives, for example, *Bacteroides thetaiotaomicron*, is known for indole and its derivatives production, such as IAA and ILA.^36^ Besides, *Anaerostipes* is well-known for producing both short-chain fatty acids (SCFA) and facilitating TRP metabolism, generating ILA.^9,37,38^ Noteworthily, in the current study, Meth withdrawal resulted in a significant reduction of indole metabolite-producing microbiota, particularly the decreased abundance of *Akkermansia*, *Bacteroides*, and *Anaerostipes* species, which presumably facilitated the reduced production of indole derivatives, contributing to the development of anxiety and depression-like behaviors.

Recently, a single-nucleus RNA-sequencing and spatial transcriptomics in female cynomolgus macaques depression model revealed that the gene expression changes associated with depressive-like behaviors mostly in microglia;^39^ In a chronic unpredictable stress (CUS) mouse model, the microglial cells undergo dramatical dynamic changes from initial proliferation and activation to dystrophy and apoptosis. These changes were particularly pronounced in the microglia and were closely linked to depression-like behaviors.^40^ Similarly, during the Meth exposure period, microglial cells displayed a persistent activated state, characterized by enlarged cell bodies and shortened, thickened dendrites, then the microglia transitioned into a dystrophic morphology, marked by reduced cell size, decreased branches, and even fragmentation, deciphering a shifting from an initial “surveillance state” to a “dystrophic” state. Consistent with our findings, a study on Meth-exposed rats exhibited significant morphological changes in microglia within the nucleus accumbens core 31 days after Meth withdrawal, characterized by a reduction in microglial cell numbers and decreased branch lengths,^41^ similarly, the microglia injury is confirmed by another study that decreased survival microglia in the hippocampus induced by Meth.^42^

After Meth exposure, the reduction of indole metabolites profoundly impacts the gut-microbiota-brain axis, a complex bidirectional communication system that relies on the AhR to transmit TRP metabolic signaling to the brain via immune response, peripheral metabolic circulation, and the vagus nerve. Noteworthily, the microbial metabolites derived from the TRP metabolic pathway can cross the blood-brain barrier(BBB), facilitating tissue-specific transport and gut-brain interactions.^43^ Similar studies have analyzed the decreased levels of IAA in the blood and cerebrospinal fluid in Alzheimer’s disease (AD) patients that the declined IAA decreases the combination of AhR, which promotes the NLRP3 inflammasome accumulation via NF-κB activation, thereby stimulating neuroinflammation.^44^ On the contrary, supplementation with indole, IS, IPA, and IAld significantly reduced brain inflammation in germ-free mice.^13^ Similarly, in SPF mice, indole and its derivatives promote neurogenesis via the AhR, while other metabolic product of TRP, such as kynurenine, exhibits no neurogenic effects.^17^ Moreover, in a mouse model of irritable bowel syndrome (IBS) induced by *Citrobacter rodentium* infection, enhanced microbial TRP metabolism by *Lactobacillus reuteri* restored colonic sensitivity and reduced anxiety-like behavior by upregulating IL-22 levels in the colon.^45^ These results highly supported the Meth-induced depression and anxiety-like behaviors involving indole metabolite-AhR signaling defects, which may provide promising therapeutic targets. Compared to other studies, our research for the first time, revealed the microbiota in Meth treated mice but not limited to the neural injury can ignite depression and anxiety-like changes. More specifically, microbiota metabolites, such as IPA and IAA, exerted salutary effects on the Meth induced neurobehavioral abnormalities, which provide a novel interventive strategy.

To ascertain the critical roles of the AhR in Meth induced neurobehavioral disorders, supplementation with indole derivatives and the AhR agonist Ficz in Meth-withdrawal mice significantly restored microglial morphology and improved neurogenesis. To further explore the specific role of AhR in the neuroprotective effects of indole derivatives, the AhR KO mice were exploited. Intriguingly, the protective effects of indole derivatives against Meth induced microglial dystrophy and neurogenesis defects were ablated in AhR KO mice, underpinning the specific salutary roles of indole derivatives in the regulation of microglial activity and neurogenesis via AhR, placing a unique role of AhR in Meth induced neurobehavioral abnormalities.

**Figure 10.**
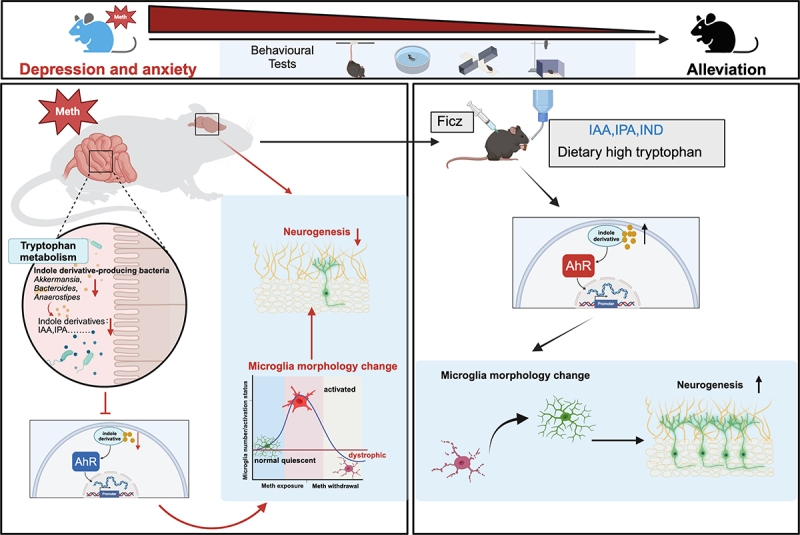
A schematic depicting the peripheral mechanism underlying Meth-induced anxiety and depression-like behaviors, particularly highlighting the role of microbiota and their indole metabolites. During Meth withdrawal, a reduction of indole metabolite-producing microbiota decreases the levels of indole metabolites, which impedes AhR expression and causes microglial dystrophy and neurogenesis defects and finalizes anxiety and depression-like behaviors in mice. Noteworthily, supplementing with indole metabolites, TRP diet, or targeting AhR could offer novel strategies for Meth-induced anxiety and depression.

In conclusion, this study, for the first time, deciphered the peripheral mechanisms by unrecognized Meth-induced anxiety and depression-like behaviors via the disturbance of microbiota and the corresponding metabolites, particularly the indole derivatives. In this process, Meth withdrawal induced decrease of indole metabolite-producing microbiota, contributing to the striking reduction of indole metabolites, which in turn, decreased AhR expression and impacted microglial morphology and neurogenesis, and finalized the anxiety and depression-like changes in mice (Figure 10). Therefore, the present study provide novel biological insights into the anxiety and depression symptoms associated with Meth withdrawal and offer potential targets and strategies for Meth abuse. Restoring the balance of the gut microbiota, particularly by promoting the synthesis of TRP metabolites such as indole derivatives, may serve as an effective intervention to alleviate Meth withdrawal symptoms. For example, the use of probiotics, dietary adjustments (e.g., high-TRP diets), or microbiota-targeting strategies to restore gut function may help reduce anxiety and depression symptoms in Meth-addicted individuals. Therefore, future drug interventions may be developed based on the regulation of the TRP microbiota metabolism and AhR receptors, like AhR agonists, which may provide innovative strategies for Meth associated neurotoxicity.

## 5. Limitation

The present study still has some limitations. Firstly, due to certain constraints, we were unable to conduct systematic anxiety and depression assessments in Meth-abusing individuals, nor were we able to obtain fecal samples from this population for further analysis of indole metabolism. Secondly, although our study demonstrates that indole derivatives confer neuroprotective effects through AhR activation, the precise mechanisms of interaction among these molecules remain unclear. AhR signaling is highly complex and context-dependent, with its effects varying across tissues and cell types. Further research is needed to elucidate the cell-specific and tissue-specific roles of AhR activation in the gut-brain axis. Thirdly, our experiments primarily relied on a mouse model to investigate Meth withdrawal and its effects on the gut microbiota and behaviors, however, they cannot totally represent the toxic effects of Meth abuse and the characteristics of mental disorders in humans. Translational studies in non-human primates or Meth-abusing human populations will be essential to confirm the clinical applicability of our findings. Fourthly, although a reduction in *Akkermansia* and its potential link to mucus layer disruption were confirmed, we did not directly demonstrate the functional contribution of this bacterial species to Meth-induced gut dysbiosis and behavioral abnormalities. Future studies using approaches such as *A. muciniphila* supplementation or depletion experiments are warranted to establish its causal role in this process. Lastly, our study focused on the role of indole derivatives and AhR activation in Meth withdrawal-related anxiety and depression. However, the development of these behaviors is multifactorial, involving other neurotransmitter systems, immune pathways, and environmental factors. Integrating these additional variables into future research will provide a more holistic understanding of Meth-induced neurobehavioral changes.

## Supplementary Material

Supplemental Material

## Data Availability

All data generated or analyzed during this study are included in this published article and its supplementary information files. The datasets presented in this study can be found in online repositories. The names of the repository/repositories and accession number(s) can be found below: https://www.ncbi.nlm.nih.gov/, PRJNA1177660.
