# Supplementary material for: Indole derivatives ameliorated the methamphetamine-induced depression and anxiety via aryl hydrocarbon receptor along “microbiota-brain” axis
Source: Gut Microbes. 2025 Feb 25;17(1):2470386. doi: 10.1080/19490976.2025.2470386 (PMC11864316; doi:10.1080/19490976.2025.2470386)
Supplement: Supplemental Material [file KGMI_A_2470386_SM5096.zip › revised Supplementary Materials .docx]

Supplementary Materials for

**Indole derivatives ameliorated the methamphetamine-induced depression and anxiety via aryl hydrocarbon receptor along “microbiota-brain” axis**

Xi Wang^a, b,1^, Miaoyang Hu ^a,1^, Weilan Wu^c^, Xinyu Lou^a^, Rong Gao^c^, Tengfei Ma^d^, S Thameem Dheen ^b^, Jie Cheng^a^, Jianping Xiong^a^, Xufeng Chen^e*^, Jun Wang^a,e*^

^a^ Center for Global Health, the Key Laboratory of Modern Toxicology, Ministry of Education, Department of Toxicology, School of Public Health, School of Public Health, Nanjing Medical University, Nanjing 211166, China

^b^ Department of Anatomy, Yong Loo Lin School of Medicine, National University of Singapore, Singapore 117594, Singapore

^c^ Department of Hygienic Analysis and Detection, the Key Laboratory of Modern Toxicology, Ministry of Education, School of Public Health, Nanjing Medical University, Nanjing 211166, China

^d^ Stem Cell and Neural Regeneration and Key Laboratory of Cardiovascular & Cerebrovascular Medicine, School of Pharmacy, Nanjing Medical University, Nanjing 211166, China

^e^ Department of Emergency Medicine, the First Affiliated Hospital of Nanjing Medical University, 300 Guangzhou Road, Nanjing, Jiangsu 210029, China

^1^ Xi Wang and Miaoyang Hu contributed equally to this work.

*Correspondence

Jun Wang, Department of Toxicology, School of Public Health, Nanjing Medical University, 818 Tianyuan East Road, Nanjing 211166, China.

E-mail Address: [wangjun@njmu.edu.cn](mailto:wangjun@njmu.edu.cn)

Xufeng Chen, Department of Emergency Medicine, the First Affiliated Hospital of Nanjing Medical University, 300 Guangzhou Road, Nanjing 210029, China

E-mail Address: [cxfyx@njmu.edu.cn](mailto:cxfyx@njmu.edu.cn)

# Method

## 1. Forced Swim Test (FST)

The forced swim test was conducted using a transparent glass swim tank with a video camera positioned in front. Each mouse was individually tested. Mice were placed in a swim tank filled with water (temperature: 23±1°C), and their behavior was recorded for 6 minutes. The total immobility time during this period was recorded. Immobility was defined as the absence of movement except for the necessary motions to keep the head above water. The proportion of immobility time was then calculated. Analysis of immobility was conducted using Superfst software (Soft-maze software Co., China).

## 2. Tail Suspension Test (TST)

In the tail suspension test, each mouse was individually tested. The tail was taped 3 cm from the tip, and the mouse was suspended with its head facing downward (approximately 15 cm from the surface). Mice typically struggle to overcome the abnormal position, but eventually exhibit intermittent immobility, indicating a state of behavioral despair. Each suspension lasted for 6 minutes, during which the total immobility time was recorded. After the test, mice were promptly removed and returned to their home cage. The ratio of immobility was quantified using Tail Suspension-scan software (Geneandi Co., China) over the observation period to assess behavioral despair.

## 3. Elevated Plus Maze (EPM)

The elevated plus maze consists of two open arms, two closed arms, and a central area. Mice were placed in the central area facing an open arm, and their behavior was recorded for 6 minutes using a video system. The time spent in the open arms and closed arms was measured. After each test, feces were removed, and the maze was sprayed with 75% ethanol and wiped with a clean cloth to prevent scent contamination from affecting subsequent tests. The movement of mice was recorded and analyzed using ANY-maze software (Stoelting Co., USA).

## 4. Open Field Test (OFT)

The open field test was conducted in a square open field box with a camera positioned above. The floor of the open field was divided into a central area and a peripheral area. Mice were placed in the central area, and their movement was recorded for 6 minutes. The number of crossings and total time spent in the central area were measured. After each test, feces were removed, and the floor of the open field box was sprayed with 75% ethanol and wiped with a clean cloth to eliminate residual odors. The time spent in different areas was recorded and analyzed using ANY-maze software (Stoelting Co., USA).

# Table S1. Primers used for RT-qPCR

| ﻿Target Gene | ﻿Primer Sequences (5’-3’) | |
| --- | --- | --- |
|  | Forward | Reverse |
| *Gapdh* | GTGGACCTCATGGCCTACAT | TGTGAGGGAGATGCTCAGTG |
| *Ahr* | GGCTTTCAGCAGTCTGATGTC | CATGAAAGAAGCGTTCTCTGGC |
| *Cyp1a1* | CAGGATGTGTCTGGTTACTTTGAC | CTGGGCTACACAAGACTCTGTCTC |
| *Cyp1b1* | CCACCAGCCTTAGTGCAGAC | GGCCAGGACGGAGAAGAGT |
| PCR① | CTTATCAGTGGGTGGAGCAATAAG | CCTTGCTATTTCAGCATGGTG |
| PCR② | CAATGGAGCTGTTTGGATGGTAC | TGCCTTATTCCCACTGAACCTG |

*The genotyping strategy involves two PCR reactions designed to differentiate between wild-type (Ahr+/+), heterozygous (Ahr+/-), and homozygous knockout (Ahr-/-) genotypes. PCR① reaction obtains a single WT band; PCR② reaction obtains a single WT band. Heterozygote: PCR① reaction obtains a WT band and a KO band; PCR② reaction obtains a WT band. Homozygote: PCR① reaction obtains a single KO band; PCR② reaction without product.

# Supplemental figures and legends


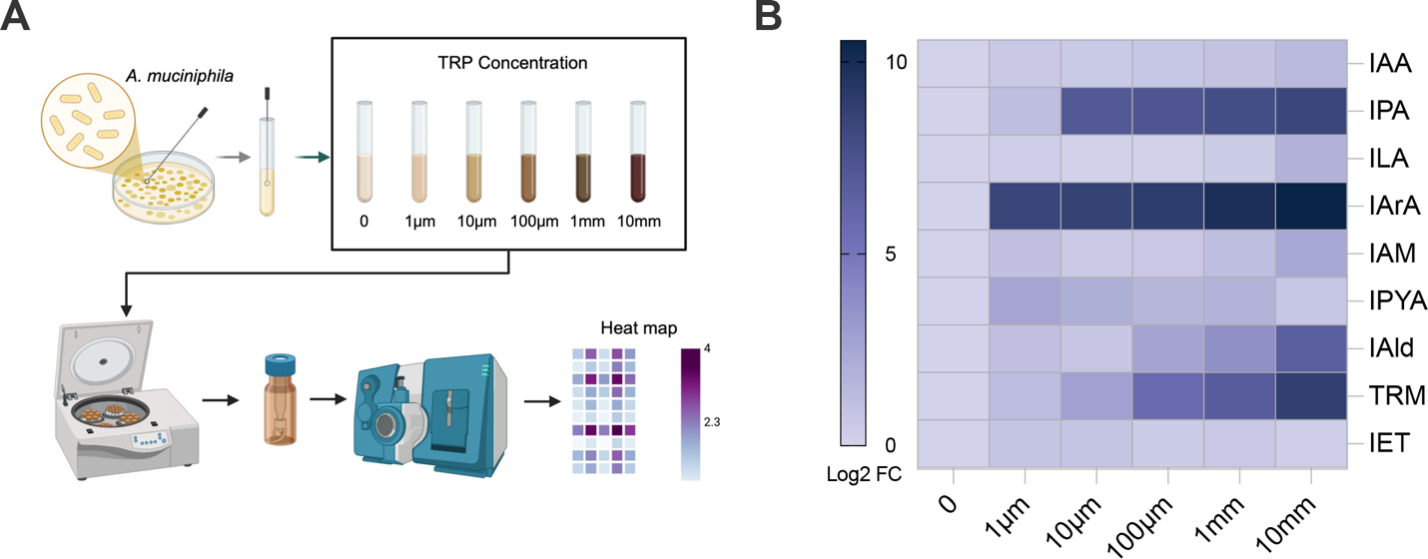


Figure S1. *A. muciniphila* metabolizes TRP into indole derivatives in a concentration-dependent manner. (A) Experimental workflow: *A. muciniphila* cultures were incubated with different concentrations of TRP (0, 1 μM, 10 μM, 100 μM, 1 mM, and 10 mM). After incubation, samples were processed and then analyzed using UHPLC-MS/MS to determine the production of indole derivatives. (B) Heatmap showing the log2 fold change (FC) of various indole derivatives produced by *A. muciniphila* at different TRP concentrations. The intensity of color indicates the level of production, with darker colors representing higher log2 FC values.


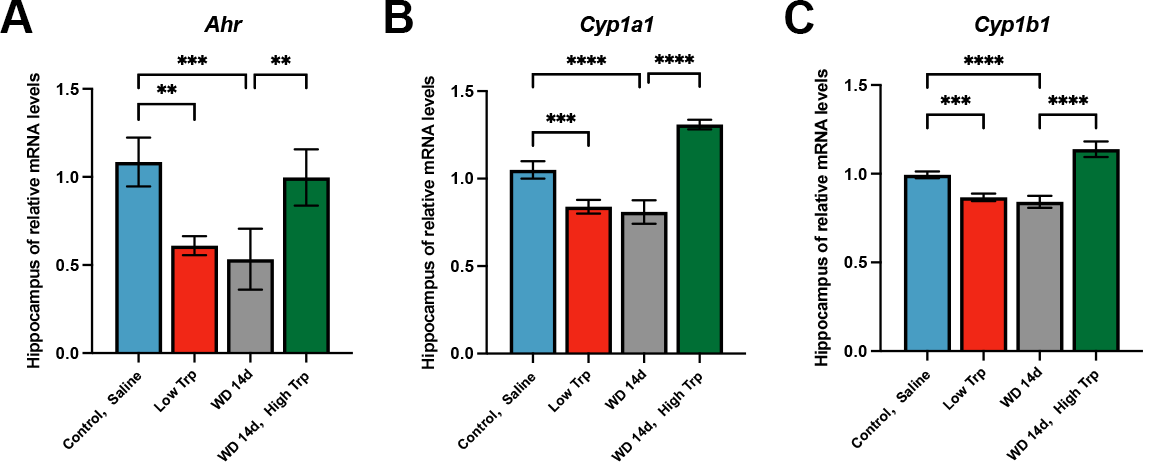


Figure S2. mRNA expression of AhR pathway-related genes (*Ahr*, *Cyp1a1*, and *Cyp1b1*) in hippocampus. Data are expressed as mean ± SEM, **p < 0.01, ***p < 0.001, ****p < 0.0001.


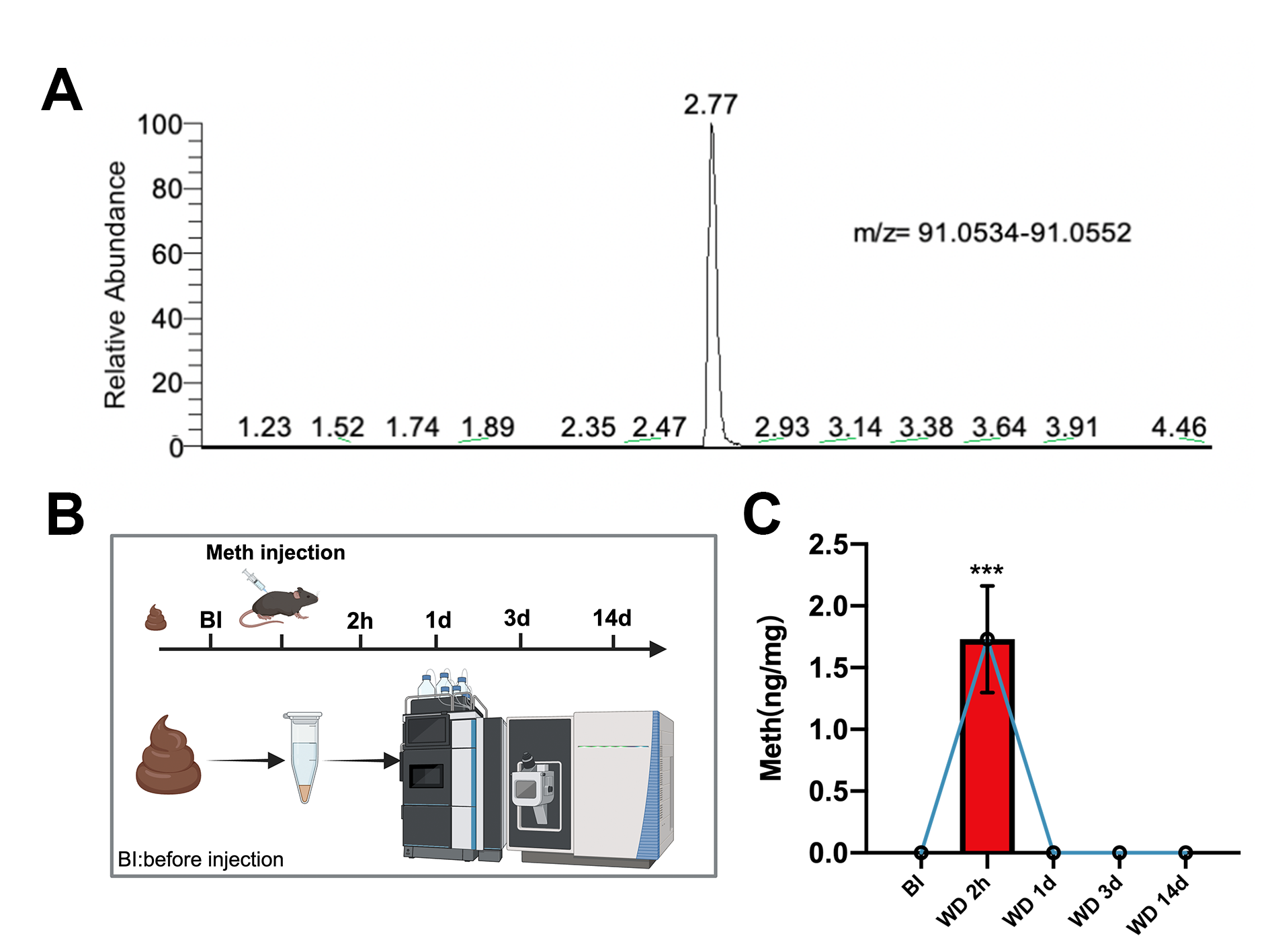


Figure S3. Detection of residual Meth in mouse feces post-injection. (A) Extracted ion chromatograms of the authentic matrix of Meth. (B) Experimental flow chart. (C) The content of meth in feces at different time points. Data are expressed as mean ± SEM, ***p < 0.001. BI: before injection.


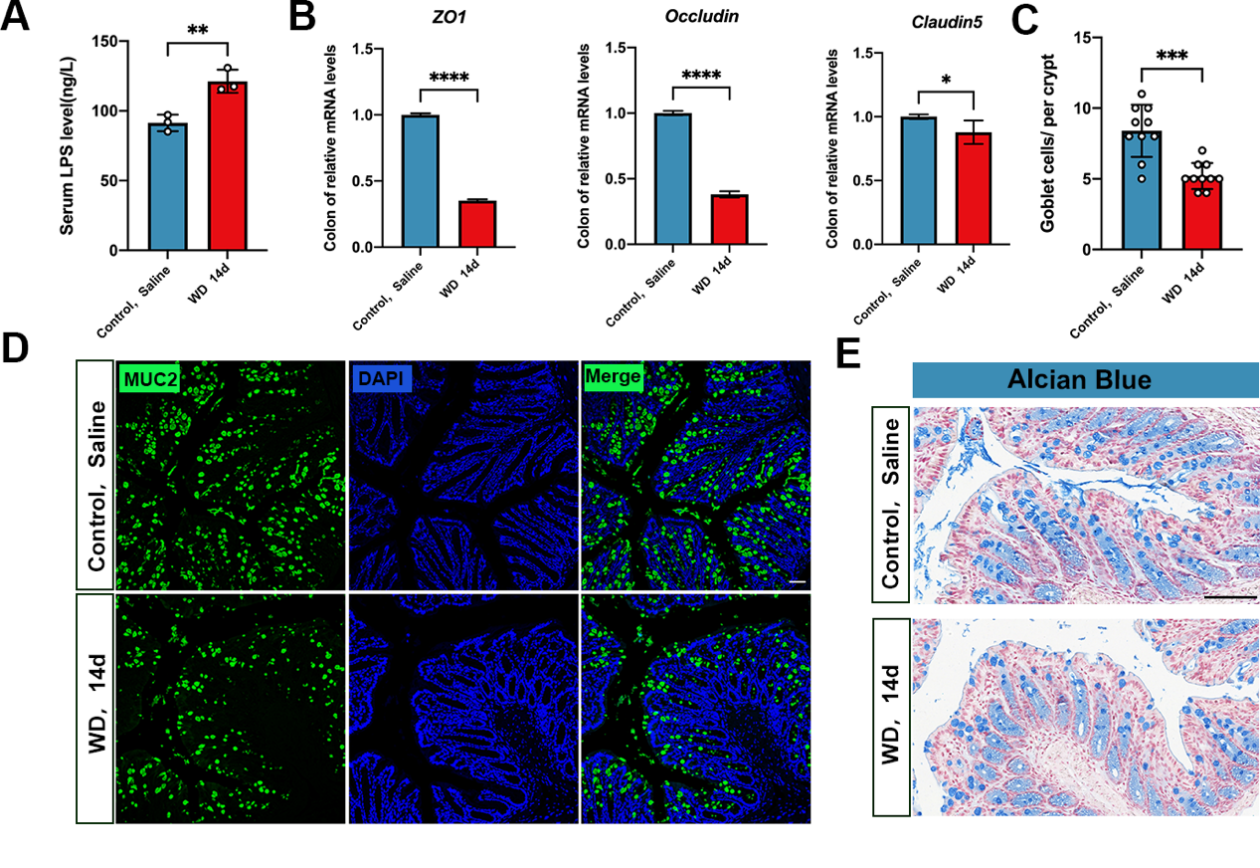


Figure S4. Meth withdrawal disrupts gut barrier integrity and mucus production. (A) Serum LPS levels. (B) mRNA levels of tight junction-related genes. (C) Goblet cell numbers per crypt. (D) Representative immunofluorescence staining images of MUC2 (green) and DAPI (blue). (E) Alcian blue staining. Data are expressed as mean ± SEM, *p < 0.05, **p < 0.01, ***p < 0.001, ****p < 0.0001.


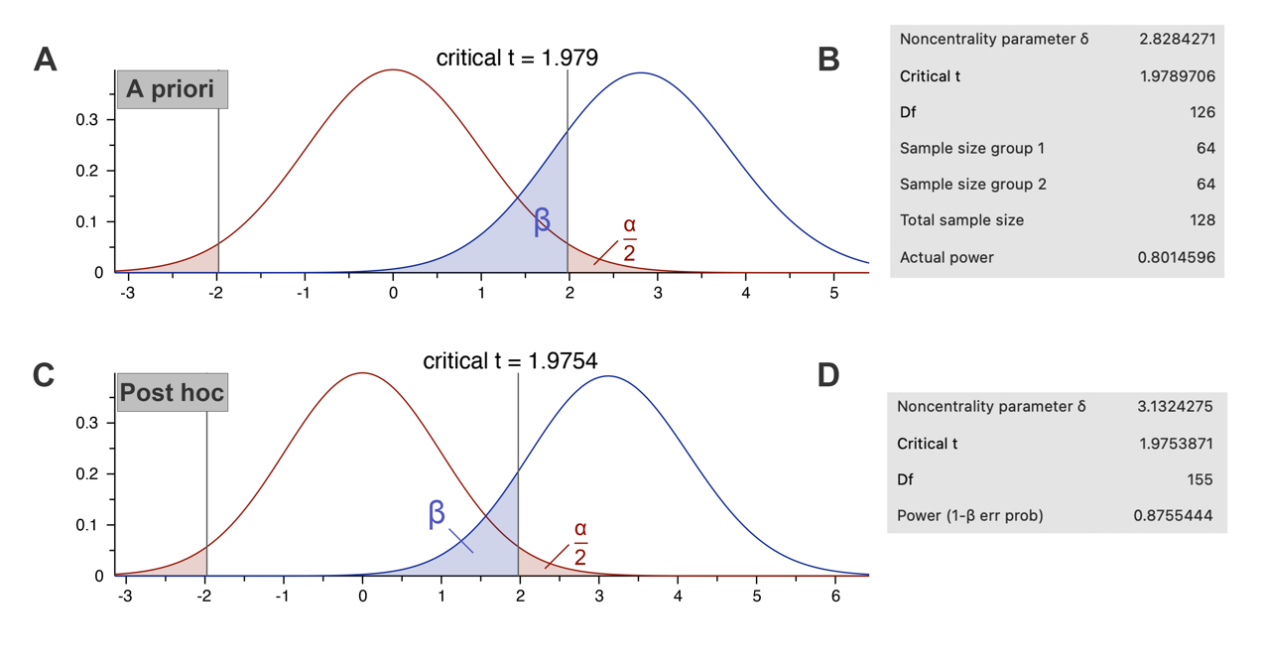


Figure S5. Power analyses for determining sample size and evaluating statistical power. (A) A priori power analysis was performed using G Power to calculate the required sample size. (B) Summary of parameters for the A priori analysis. (C) Post hoc power analysis was conducted to evaluate the statistical power of the current sample size (78 Meth abusers and 79 healthy controls). (D) Summary of parameters for the Post hoc analysis.
